# A complete enzymatic capacity for biosynthesis of docosahexaenoic acid (DHA, 22 : 6n–3) exists in the marine Harpacticoida copepod *Tigriopus californicus*

**DOI:** 10.1098/rsob.200402

**Published:** 2021-04-28

**Authors:** Naoki Kabeya, Masanari Ogino, Hideki Ushio, Yutaka Haga, Shuichi Satoh, Juan C. Navarro, Óscar Monroig

**Affiliations:** ^1^ Department of Marine Biosciences, Tokyo University of Marine Science and Technology, Konan 4-5-7, Minato, Tokyo, Japan; ^2^ Department of Aquatic Bioscience, The University of Tokyo, Yayoi 1-1-1, Bunkyo, Tokyo, Japan; ^3^ Instituto de Acuicultura Torre de la Sal (IATS-CSIC), Ribera de Cabanes 12595, Castellón, Spain

**Keywords:** biosynthesis, polyunsaturated fatty acids, fatty acyl elongases, front-end desaturases, methyl-end desaturases, harpacticoid copepods

## Abstract

The long-standing paradigm establishing that global production of Omega-3 (n–3) long-chain polyunsaturated fatty acids (LC-PUFA) derived almost exclusively from marine single-cell organisms, was recently challenged by the discovery that multiple invertebrates possess methyl-end (or *ω*x) desaturases, critical enzymes enabling the biosynthesis of n–3 LC-PUFA. However, the question of whether animals with *ω*x desaturases have complete n–3 LC-PUFA biosynthetic pathways and hence can contribute to the production of these compounds in marine ecosystems remained unanswered. In the present study, we investigated the complete enzymatic complement involved in the n–3 LC-PUFA biosynthesis in *Tigriopus californicus*, an intertidal harpacticoid copepod. A total of two *ω*x desaturases, five front-end desaturases and six fatty acyl elongases were successfully isolated and functionally characterized. The *T. californicus ω*x desaturases enable the *de novo* biosynthesis of C_18_ PUFA such as linoleic and α-linolenic acids, as well as several n–3 LC-PUFA from n–6 substrates. Functions demonstrated in front-end desaturases and fatty acyl elongases unveiled various routes through which *T. californicus* can biosynthesize the physiologically important arachidonic and eicosapentaenoic acids. Moreover, *T. californicus* possess a Δ4 desaturase, enabling the biosynthesis of docosahexaenoic acid via the ‘Δ4 pathway’. In conclusion, harpacticoid copepods such as *T. californicus* have complete n–3 LC-PUFA biosynthetic pathways and such capacity illustrates major roles of these invertebrates in the provision of essential fatty acids to upper trophic levels.

## Graphical abstract

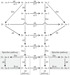

## 1. Introduction

**Figure 1.**
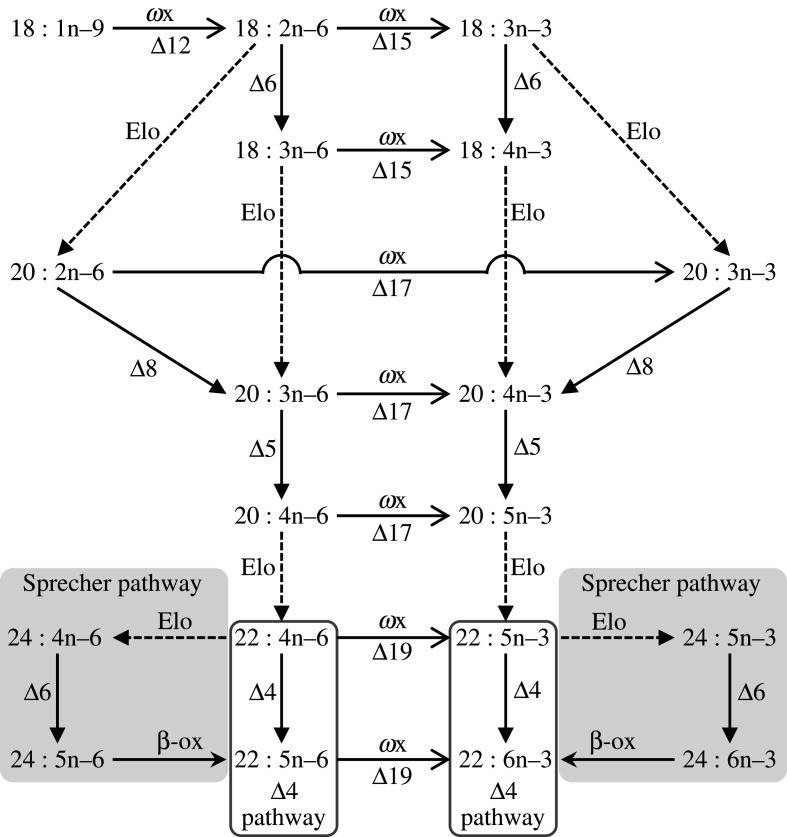
A general illustration of the PUFA and LC-PUFA biosynthetic pathways. Reactions catalysed by front-end desaturases are indicated as Δ6, Δ8, Δ5 and Δ4. Reactions catalysed by ωx desaturases are indicated as ‘*ω*x’ with their corresponding Δ regioselectivity indicated underneath. Elongase-mediated reactions are denoted as ‘Elo’.

The omega-3 (*ω*3 or n–3) long-chain (≥C_20_) polyunsaturated fatty acids (LC-PUFA) including eicosapentaenoic acid (EPA, 20 : 5n–3) and docosahexaenoic acid (DHA, 22 : 6n–3) have beneficial effects on human health [1,2]. Marine ecosystems have been regarded to be responsible for virtually all the global production of n–3 LC-PUFA due to the abundance of single-cell microorganisms such as photosynthetic microalgae, heterotrophic protists and bacteria, with the ability to biosynthesize n–3 LC-PUFA [3–5]. The biosynthesis of n–3 LC-PUFA in marine microbes is achieved by either anaerobic or aerobic pathways involving distinct enzymatic machineries. The polyketide synthase (PKS) complex is involved in the anaerobic pathway existing in prokaryotes and some eukaryotic microorganisms [6]. However, most eukaryotes operate the aerobic pathway of n–3 LC-PUFA biosynthesis that entails two critical components; one is the biosynthesis of n–3 C_18_ polyunsaturated fatty acid (PUFA), namely α-linolenic acid (ALA, 18 : 3n–3), from saturated fatty acids (SFA) such as stearic acid (18 : 0); another is the biosynthesis of n–3 LC-PUFA including EPA and DHA from ALA (figure 1). Along with the aerobic n–3 LC-PUFA biosynthetic pathways, a varied range of enzymes catalyses multi-step desaturation (introduction of new double bonds or unsaturations) and elongation (extension of the carbon acyl chain) reactions. Initially, palmitic acid (16 : 0) is synthesized *via* the fatty acid synthase (FAS) system and then elongated to 18 : 0. Subsequently, a Δ9 desaturase (e.g. stearoyl-CoA desaturase) introduces the first double bond to produce the monounsaturated fatty acid (MUFA) oleic acid (18 : 1n–9) [7]. These enzymatic capacities enabling the biosynthesis of SFA and MUFA are virtually present in all eukaryotes [8,9]. However, enzymes enabling the *de novo* biosynthesis of PUFA (i.e. introducing a second double bond into oleic acid) have a more restricted distribution and, as discussed below, were believed to be largely absent from animals. Specifically, the *de novo* biosynthesis of PUFA typically requires the action of a particular type of desaturase enzymes called methyl-end (or *ω*x) desaturases since they introduce a new double bond between a pre-existing one and the methyl-terminus of the carbon chain [10]. Thus, there exist *ω*x desaturases that introduce a new double bond into oleic acid at Δ12 position producing the PUFA linoleic acid (LA, 18 : 2n–6). Others introduce a further double bond into LA at Δ15 position and thus produce the n–3 C_18_ PUFA ALA (figure 1). Both LA and ALA become substrates from which n–6 and n–3 LC-PUFA, respectively, can be biosynthesized through sequential reactions catalysed by front-end desaturases, which introduce a new double bond between the pre-existing one and the carboxyl-terminus of the fatty acid, and fatty acyl elongases, which catalyse the initial condensation step of the fatty acid elongation pathway (figure 1) [11,12]. Animals possess a varied complement of front-end desaturases and fatty acyl elongases enabling them to produce LC-PUFA from the C_18_ PUFA precursors LA and ALA [10,13].

Challenging the widely accepted dogma establishing that, with few exceptions (e.g. [14–17]), animals lack the ability to biosynthesize PUFA *de novo*, Kabeya *et al*. [18] demonstrated that a plethora of invertebrates including cnidarians, nematodes, lophotrochozoans and arthropods possess *ω*x desaturases enabling them to biosynthesize PUFA *de novo*. Importantly, it was further established that invertebrates also have *ω*x desaturases commonly known as ‘*ω*3 desaturases’, which enable conversions of multiple n–6 fatty acids into the corresponding n–3 desaturated products including LC-PUFA such as EPA and DHA [18–20]. These findings have obvious ecological implications associated with the contribution that these invertebrates can have on the primary production of the essential compounds n–3 LC-PUFA at a global scale. This is particularly true for groups that are abundant and widely distributed in marine ecosystems.

Copepods play critical roles in the trophic ecology of marine ecosystems not only because they are the most abundant zooplanktonic crustaceans but also because they represent key prey items ensuring the transfer of essential nutrients to upper trophic level organisms. Indeed, copepods contain high levels of n–3 LC-PUFA, particularly EPA and DHA [21], and thus these crustaceans become critically important to guarantee the provision of these essential nutrients to high trophic level organisms such as fish [22]. While the abovementioned abundance of LC-PUFA in primary producers suggests that the high LC-PUFA content of copepods' lipids can have a dietary origin, it is unclear to what extent copepods can modulate their own fatty acid profiles and contribute to their characteristic high LC-PUFA lipid profiles. Early studies using ^14^C-labelled fatty acids suggested some copepods showed the ability to bioconvert PUFA into LC-PUFA [23,24]. Studies involving feeding trials using LC-PUFA-deficient diets or stable isotope labelled fatty acids provided further evidence that Harpacticoida and Cyclopoida copepods possess some capacity to produce n–3 LC-PUFA endogenously [25–33]. However, these studies could not unequivocally establish that the abovementioned metabolic activities observed were indeed due to the copepod's enzymatic complement, and hence it is difficult to completely rule out that LC-PUFA are rather synthesized by microbes that coexist within the copepod. More robust evidence has been recently collected when genes encoding *ω*x desaturases were found in several species from groups of copepods including Cyclopoida, Harpacticoida and Siphonostomatoida [18]. However, with the sole exception of *Lepeophtheirus salmonis* (Siphonostomatoida), none of copepod *ω*x desaturases has been functionally characterized yet, and therefore, it remains unknown whether the activities of the copepod *ω*x desaturases are conserved across the group or, on the contrary, have diversified during evolution as occurred for other fatty acyl desaturases [13]. In addition, it is unclear whether, beyond *ω*x desaturases, copepods have further enzymes such as front-end desaturases and fatty acyl elongases that, along *ω*x desaturases, enable complete enzymatic activities allowing the production of a variety of LC-PUFA including DHA (figure 1). Recent studies involving genomic and transcriptomic analyses identified sequences of desaturase and elongase genes with putative roles in the LC-PUFA biosynthesis in Harpacticoida and Cyclopoida copepods [31–34]. However, no functional evidence demonstrating the actual role of these enzymes in the LC-PUFA biosynthetic pathways was reported.

The marine intertidal Harpacticoida copepod *Tigriopus californicus* has been used as a model organism for the study in several disciplines including ecology, evolution and genetics [35–37]. In this study, we investigated the LC-PUFA biosynthetic capability of *T. californicus* by performing a comprehensive search for genes encoding putative *ω*x desaturases, front-end desaturases and fatty acyl elongases in *T. californicus* transcriptomic and genomic datasets. A total of 13 genes encoding two *ω*x desaturases, five front-end desaturases and six fatty acyl elongases were successfully identified. We further cloned and functionally characterized the 13 *T. californicus* desaturases and elongases by heterologous expression in yeast demonstrating that *T. californicus* has a comprehensive enzymatic set enabling the endogenous production of LC-PUFA including EPA and DHA. These results suggest that harpacticoid copepods such as *T. californicus* can be a net producer of the physiologically important n–3 LC-PUFA in marine supralittoral ecosystems.

## 2. Material and methods

### 2.1. RNA extraction and cDNA synthesis

Live *T. californicus* were purchased from Reefphyto Ltd. (Newport, UK). Total RNA was extracted from pooled (approx. 50) whole individuals using TRI Reagent (Sigma-Aldrich, Dorset, UK) following the manufacturer's recommendations. The extracted RNA was treated with RQ1 RNase-Free DNase (Promega, Madison, WI, USA) to remove potential genomic DNA contamination. Subsequently, the complementary DNA (cDNA) was synthesized from 2 μg of total RNA using SuperScript III First-Strand Synthesis System for RT-PCR (Thermo Fisher Scientific, Waltham, MA, USA). The obtained cDNA was stored at −20°C until further use. All experiments were carried out in compliance with the guidelines for the care and use of laboratory animals of the Tokyo University of Marine Science and Technology.

### 2.2. Isolation of putative desaturase and elongase genes from *Tigriopus californicus*

In addition to the two *ω*x desaturase sequences identified by Kabeya *et al*. [18], termed herein as *ωx1* (NCBI acc. No. JW524768) and *ωx2* (GBTC01004759), further genes encoding PUFA biosynthesizing enzymes including putative front-end desaturases and fatty acyl elongases were retrieved from the *T. californicus* transcriptome shotgun assembly (NCBI BioProject No. PRJNA158547 and PRJNA263967) by DELTA-BLAST. Several functionally characterized front-end desaturases and fatty acyl elongases from invertebrates including molluscs and echinoderms were used as queries [38–43]. The DELTA-BLAST search allowed the identification of a total of 21 front-end desaturase-like and 31 fatty acyl elongase-like sequences that were subsequently assembled to generate several consensus sequences. As a result, full-length open reading frames (ORF) of five putative front-end desaturases and five putative elongases were obtained, which were named as *Fed1* to *Fed5* and *Elo1* to *Elo5*, respectively. After this search, a new transcriptomic dataset became available at NCBI (No. PRJNA504307). Thus, the same search against the new database was repeated and an additional elongase-like sequence was successfully obtained, which was named as *Elo6*. We then performed *blastn* search against *T. californicus* genome assembly (https://i5k.nal.usda.gov/Tigriopus_californicus) and confirmed all five front-end desaturase and six fatty acyl elongase sequences were successfully found in the *T. californicus* genome. Apart from the *T. californicus ωx1* (JW524768) and *ωx2* (GBTC01004759) found by Kabeya *et al*. [18], no further *ω*x desaturases were identified in the *T. californicus* genome.

### 2.3. Phylogenetic analysis

Phylogenetic trees comparing the deduced amino acid (aa) sequences of front-end desaturases and fatty acyl elongases from a wide range of eukaryotic organisms were constructed using the maximum-likelihood (ML) method [44]. A comprehensive phylogenetic analysis of *ω*x desaturases including the two of *T. californicus* was carried out by Kabeya *et al*. [18] and hence, it was not performed in the present study. The sequence dataset for the ML phylogenetic analysis of front-end desaturases was built through the following steps: (i) retrieval of all eukaryotic aa sequences from RefSeq-specific protein-containing ‘Delta6-FADS-like’ domain (cd03506); (ii) selection of representative species from each taxonomic group; (iii) addition of functionally characterized genes and copepod genes and (iv) generation of multiple sequence alignment (MSA) using MAFFT v. 7 [45] with FFT-NS-i method (*--reorder --auto*), and removal of all duplicated sequences and incomplete gappy sequences. A similar strategy was applied for elongases as follows: (i) retrieval of all metazoan aa sequences from RefSeq-specific protein-containing ‘ELO’ domain (pfam01151); (ii) and (iii) were the same as above and (iv) generation of cleaned MSA as described above and removal of potential non-PUFA elongase sequences. Each dataset was then aligned using MAFFT v. 7 [45] with E-INS-i method (*--genafpair --maxiterate* 1000) and filtered through using GUIDANCE v. 2.0 [46] with 100 bootstrap replicates, sequence and column masking cut-off threshold less than 0.5. Subsequently, the resulting MSA were filtered to delete columns containing gaps that were greater than 95% of the sequences by TrimAl [47]. The final MSA contained 254 columns and 222 sequences for front-end desaturases, and 229 columns and 184 sequences for elongases. The ML phylogenetic inference was carried out using RAxML-NG with automatic bootstrapping (*--all*) [48]. The protein substitution model was selected as LG + I + G4 for both gene types by ModelTest-NG (*--model LG* *+* *I* *+* *G4*) [49]. The resulting trees were visualized using Interactive Tree of Life (iTOL v. 5, https://itol.embl.de).

### 2.4. Functional characterization of the *Tigriopus californicus* desaturases and elongases

The full-length ORF of the *T. californicus ω*x desaturases, front-end desaturases and fatty acyl elongases were amplified by polymerase chain reactions (PCR) using a high-fidelity DNA polymerase (PrimeSTAR Max DNA polymerase, Takara Bio Inc., Shiga, Japan). All PCR runs were carried out following the manufacturer's default recommendations as follows: 10 s at 98°C for the initial denaturation, 35 cycles of 10 s at 98°C, 5 s at 55°C and 20 s at 72°C. The primer sequences containing restriction enzyme sites to enable further cloning into the yeast expression vector pYES2 (Thermo Fisher Scientific) are given in table 1. The resulting PCR products were purified from an agarose gel (GenElute Gel Extraction Kit, Sigma-Aldrich Japan K.K., Tokyo, Japan) and subsequently digested with the corresponding restriction enzymes (table 1). Next, the purified and digested full-length ORF were ligated into a similarly restricted pYES2 using T4 DNA ligase (Promega) and transformed into DH5*α* competent *E. coli* (Nippon Gene Co., Ltd., Tokyo, Japan). Positive transformant colonies were grown overnight in LB broth, and plasmid preparations (GenElute Plasmid Miniprep Kit, Sigma-Aldrich Japan K.K.) were sent to the DNA sequencing service (Eurofin Genomics K.K., Tokyo, Japan) to confirm their sequences. Plasmid constructs of the corresponding *T. californicus* two *ω*x desaturases, five front-end desaturases and six fatty acyl elongases were individually transformed into INV*Sc*1 yeast *Saccharomyces cerevisiae* (Life Technologies Japan, Tokyo, Japan) using the *S.c.* EasyComp yeast transformation kit (Life Technologies Japan). After growing the transformed yeast on *S. cerevisiae* minimal medium minus uracil (SCMM^−ura^) plates for 3 days at 30°C, one of the successful transformants from each gene was selected and used for the functional characterization assay as described below.

**Table 1.** Primer sequences for the ORF amplification of each gene. Underlined nucleotides indicate the corresponding restriction sites in each primer sequence.

target gene	NCBI accession no.	sense primer		antisense primer	
		name	sequence	name	sequence
*ω*x1	MT757172	TcWx1_HindIII_F	5'-CCCAAGCTTACCATGAGTCCCAATTCCTC-3'	TcWx1_XbaI_R	5'-CCGTCTAGATTACAATGATTTGTTTGAGAGCGTA-3'
*ω*x2	MT757173	TcWx2_HindIII_F	5'-CCCAAGCTTACTATGGCTTCCGATTATACAG-3'	TcWx2_XbaI_R	5'-CCGTCTAGACTACCTTGACTTCTTAGATGA-3'
Fed1	MT757167	TcFed1_BamHI_F	5'-CCCGGATCCAAGATGTCCGCTACAAAATTAGC-3'	TcFed1_XbaI_R	5'-CCGTCTAGATTAGGAAATGTTTCTGACAATTCG-3'
Fed2	MT757168	TcFed2_KpnI_F	5'-CCCGGTACCATCATGCCTTCAAGGGAAATG-3'	TcFed2_SacI_R	5'-CCGGAGCTCCTAATTTACGAGCAGGGTCTTG-3'
Fed3	MT757169	TcFed3_SacI_F	5'-CCCGAGCTCAGAATGGCGAAGGAAAGACGTGTG-3'	TcFed3_XbaI_R	5'-CCGTCTAGATCATCCAGCCACGCTCAGGGTTC-3'
Fed4	MT757170	TcFed4_HindIII_F	5'-CCCAAGCTTAAAATGCCTGACGTTGGA-3'	TcFed4_XbaI_R	5'-CCGTCTAGATCAACCACTTAATGAGAC-3'
Fed5	MT757171	TcFed5_HindIII_F	5'-CCCAAGCTTAAAATGGCGCCAAACGCAAC-3'	TcFed5_XbaI_R	5'-CCGTCTAGATTATGACGTGACGAGATGCTTGG-3'
Elo1	MT757162	TcElo1_HindIII_F	5'-CCCAAGCTTAGCATGGACTTTCTCGTGGA-3'	TcElo1_XbaI_R	5'-CCGTCTAGATTATTCCTTGTGGGCTTTCGA-3'
Elo2	MT757163	TcElo2_HindIII_F	5'-CCCAAGCTTACCATGGGTTCCCTGATACA-3'	TcElo2_XbaI_R	5'-CCGTCTAGATCAAGCGCTCTTTCTGGATTTG-3'
Elo3	MT757164	TcElo3_HindIII_F	5'-CCCAAGCTTAACATGTCGGTGGTGGATTTG-3'	TcElo3_XbaI_R	5'-CCGTCTAGACTAATCTTCCTTGATCATCGTGGC-3'
Elo4	MT757165	TcElo4_HindIII_F	5'-CCCAAGCTTAACATGGAGCTTCTGGACGAT-3'	TcElo4_XbaI_R	5'-CCGTCTAGATTATTGGCGTTTCTGGTTCTCG-3'
Elo5	MT757166	TcElo5_BamHI_F	5'-CCCGGATCCAAGATGAACCGAATTCTGGAGGA-3'	TcElo5_XhoI_R	5'-CCGCTCGAGTCATTGAGATTTGGTGGCCAAG-3'
Elo6	MW246081	TcElo6_HindIII_F	5'-CCCAAGCTTACCATGAACACATTGGAAGC-3'	TcElo6_XbaI_R	5'-CCGTCTAGATCAGTTATTCTTGTCATATCTTTGAAGC-3'

Each obtained transformant was individually grown in SCMM^−ura^ broth and diluted to OD_600_ = 0.4 in one single Erlenmeyer flask for each potential substrate assayed. After the OD_600_ reached 1, the cultures were supplemented with 2% (w/v) galactose for the induction of transgene expression as well as with one of the potential PUFA substrates for each enzyme [10]. For *ω*x desaturases, the exogenously supplied PUFA were 18 : 2n–6, 18 : 3n–6, 20 : 2n–6, 20 : 3n–6, 20 : 4n–6, 22 : 4n–6 and 22 : 5n–6. For front-end desaturases, PUFA substrates included 18 : 2n–6, 20 : 2n–6, 20 : 3n–6, 22 : 4n–6, 18 : 3n–3, 20 : 3n–3, 20 : 4n–3 and 22 : 5n–3, and for elongases exogenously supplied PUFA substrates were 18 : 2n–6, 18 : 3n–6, 20 : 4n–6, 22 : 4n–6, 18 : 3n–3, 18 : 4n–3, 20 : 5n–3 and 22 : 5n–3. Since certain *ω*x desaturases have shown the capacity to desaturate yeast endogenous FA [18–20], transgenic yeast expressing the two *T. californicus ω*x desaturases were grown in triplicate Erlenmeyer flasks in the absence of exogenously added FA substrates and their FA profiles compared with those of control yeast transformed with empty pYES2 vector (also *n* = 3). Additionally, to test Δ6 desaturase activity towards 24 : 5n–3, transgenic yeast co-expressing the zebrafish *elovl2* [50] and each *T. californicus fed* gene were grown in the presence of 22 : 5n–3 following the method established by Oboh *et al*. [51]. Each FA substrate was supplemented as sodium salts at concentrations of 0.5 mM (C_18_), 0.75 mM (C_20_) and 1.0 mM (C_22_) to compensate for the reduced uptake efficiency with the length of the carbon chain [19]. After 48 h of incubation at 30°C and vigorous shaking, yeast cells were harvested by centrifugation (2 min, 500 × *g*), washed twice in double distilled water, and lyophilized prior to preparation of fatty acid methyl ester (FAME) derivatives. FAME was prepared using Fatty Acid Methyl Ester Preparation Kit (Nacalai Tesque, Kyoto, Japan) following the manufacturer's recommendations. All fatty acids were purchased from Nu-Chek Prep, Inc. (Elysian, MN, USA), except 18 : 4n–3 from Larodan AB (Solna, Sweden) and 20 : 4n–3 from Cayman Chemicals (Ann Arbor, MI, USA).

### 2.5. Fatty acid analysis by gas chromatography

FAME samples were injected on a GC-2025 (Shimadzu Corporation, Kyoto, Japan) gas chromatograph equipped with a capillary column (Supelcowax 10, 30 m × 0.32 mm i.d. × 0.25 µm, Sigma-Aldrich) and a flame ionization detector (FID). The temperature conditions consisted of an initial ramp from 50°C to 180°C at a rate of 40°C min^−1^, 180°C to 230°C at a rate of 1°C min^−1^ and then 230°C for 10 min. The injection port and FID temperature were 250°C and 280°C, respectively. Helium was used as a carrier gas in constant velocity mode (30 cm sec^−1^). FAME was identified by the comparison of their retention times with those from commercial FAME standards. The conversion efficiency of three types of assayed enzymes towards the exogenously supplied PUFA substrates was calculated with the formula (all product areas/(all product areas + substrate area)) × 100 [20].

### 2.6. Statistical analysis

Comparisons of means of FA composition between control yeast (*n* = 3) and transgenic yeast expressing the *T. californicus ω*x desaturases (*n* = 3) were carried out using Dunnett's test with *p* < 0.05 indicating statistical significance. The analysis was performed in R 3.4.1. (www.r-project.org).

## 3. Results

### 3.1. Phylogeny of the *Tigriopus californicus* front-end desaturases and fatty acyl elongases

**Figure 2.**
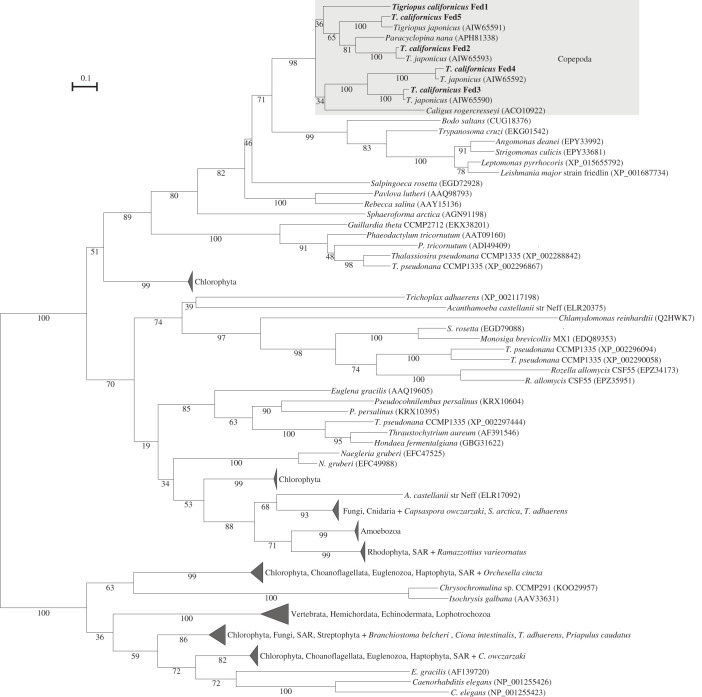
Maximum-likelihood phylogenetic analysis of the *T. californicus* front-end desaturases. The tree was visualized using iTOL (https://itol.embl.de) and re-rooted at the midpoint.

The ML phylogenetic tree of the front-end desaturases retrieved from *T. californicus* and other organisms is shown in figure 2 (a complete view is shown in electronic supplementary material, figure S1). All five *T. californicus* front-end desaturases (termed Fed1–5, figure 2) clustered together (bootstrap = 98%) with other copepod sequences from *Tigriopus japonicus* (Harpacticoida), *Caligus rogercresseyi* (Siphonostomatoida) and *Paracyclopina nana* (Cyclopoida). A direct sister group of the copepod clade could not be clearly identified due to insufficient resolution of the tree. However, the copepod clade clustered (bootstrap = 82%) with several taxonomically unrelated species belonging to the classes Kinetoplastea (e.g. *Bodo saltans*, *Trypanosoma cruzi*), Choanoflagellata (*Salpingoeca rosetta*) and Haptophyta (*Rebecca salina* and *Pavlova lutheri*). This clade was further out-grouped with another containing sequences from *Sphaeroforma arctica* (Ichthyosporea), *Guillardia theta* (Cryptophyceae) and two Bacillariophyta diatoms, namely *Phaeodactylum tricornutum* and *Thalassiosira pseudonana*. Interestingly, front-end desaturases from other metazoan species including Vertebrata, Echinodermata and Mollusca grouped together in a clearly separated clade (bootstrap = 100%) from that containing the copepod sequences (figure 2). In addition, Fat-3 (Δ6 desaturase, NP_001255426) and Fat-4 (Δ5 desaturase, NP_001255423) genes from the nematode *Caenorhabditis elegans* were also distantly located from the Copepoda clade (figure 2).

**Figure 3.**
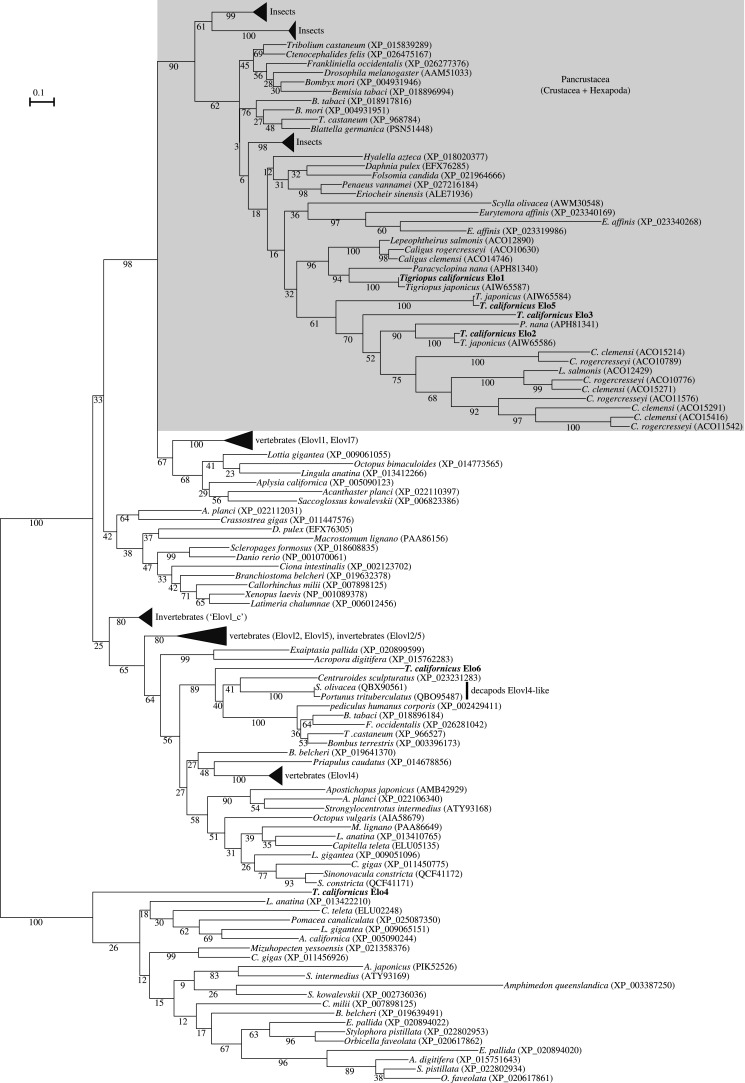
Maximum-likelihood phylogenetic analysis of the *T. californicus* fatty acyl elongases. The tree was visualized by using iTOL (https://itol.embl.de) and re-rooted at the midpoint.

The six elongases (Elo1–6) from *T. californicus* were separated to three different clades in the ML phylogenetic tree (figure 3; electronic supplementary material, figure S2). The Elo1, Elo2, Elo3 and Elo5 were branched to a Pancrustacea (=Crustacea + Hexapoda)-specific elongase clade with a relatively high support value (90%). Despite its direct sister group could not be clearly established, this Pancrustacea-specific clade grouped itself with the vertebrate Elovl1 and Elovl7 cluster (bootstrap = 98%) (figure 3). Within the Pancrustacea clade, the relationship among each taxonomical group was not well-resolved but, along Copepoda (*T. californicus*, *T. japonicus*, *P. nana*, *C. rogercresseyi*, *Caligus clemensi*, *Lepeophtheirus salmonis* and *Eurytemora affinis*), it included several insects (e.g. *Drosophila melanogaster*, *Bombyx mori*), a collembola (*Folsomia candida*), and crustaceans such as Decapoda (*Scylla olivacea*, *Eriocheir sinens*is and *Penaeus vannamei*), Branchiopoda (*Daphnia pulex*) and Amphipoda (*Hyalella azteca*) (figure 3). Interestingly, the *T. californicus* Elo1, Elo2 and Elo5 grouped with sequences from other copepods, particularly *T. japonicus*, while the relationship of the *T. californicus* Elo3 with other copepod genes could not be established (figure 3). Away from the Pancrustacea clade described above, the *T. californicus* Elo6 clustered within another well-supported clade (89%) containing several Arthropoda sequences including functionally characterized Elovl4-like sequences from two decapods (*S. olivacea* and *Portunus trituberculatus*) [52,53] (figure 3). The *T. californicus* Elo4 was grouped in a completely distinct clade that out-grouped all other sequences (figure 3). This clade was comprised a wide range of metazoan sequences except bony vertebrates. The *T. californicus* Elo4 was the only gene from Ecdysozoa species identified in our searches.

### 3.2. Functional characterization of the *Tigriopus californicus* methyl-end desaturases

**Figure 4.**
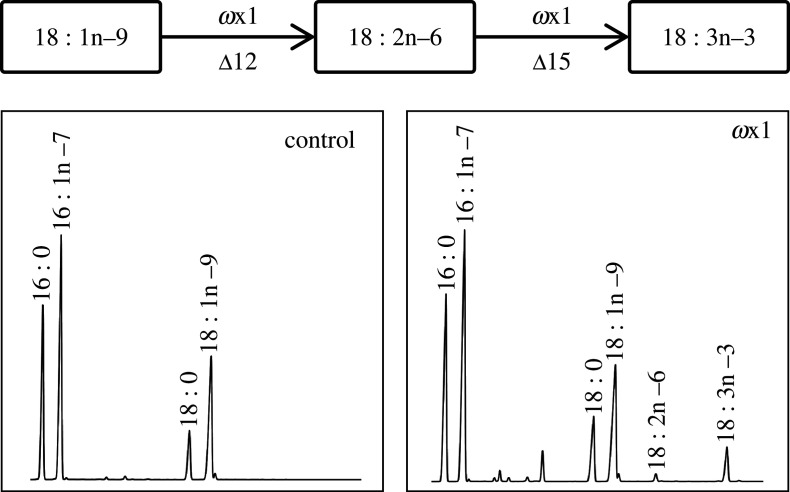
Functional analysis of the *T. californicus ω*x1 from demonstrating *de novo* PUFA biosynthesis from 18 : 1n–9. Δy in the pathway indicates the specific carbon position at which the incipient double bond locates from the front end of the fatty acyl chain.

In order to fully elucidate the functions of the *T. californicus ω*x desaturases, we first tested the desaturase activities towards yeast endogenous fatty acids. The fatty acid profile of the control yeast transformed with the empty pYES2 vector showed four prominent peaks corresponding to 16 : 0, 16 : 1 isomers, 18 : 0 and 18 : 1n–9 as previously reported for wild-type *S. cerevisiae* [54] (figure 4 and table 2). Two additional peaks corresponding to 18 : 2n–6 and 18 : 3n–3 were detected in the yeast transformed with *ωx1*, denoting Δ12 (conversion of 18 : 1n–9 to 18 : 2n–6) and Δ15 (conversion of 18 : 2n–6 to 18 : 3n–3) desaturase activities (figure 4 and table 2). These results indicate that the *T. californicus ω*x1 is a Δ12Δ15 desaturase. No additional peaks were observed in the yeast transformed with *ωx2* (table 2). Next, we tested further activities of the *T. californicus ω*x desaturases by growing the transgenic yeast containing their ORF in the presence of exogenously added n–6 PUFA substrates, namely 18 : 2n–6, 18 : 3n–6, 20 : 2n–6, 20 : 3n–6, 20 : 4n–6, 22 : 4n–6 and 22 : 5n–6. Yeast expressing the *T. californicus ωx1* converted the C_18_ and C_20_ n–6 PUFA substrates (18 : 2n–6, 18 : 3n–6, 20 : 2n–6, 20 : 3n–6 and 20 : 4n–6) but not the C_22_ (22 : 4n–6 and 22 : 5n–6), into the corresponding n–3 products (table 3; electronic supplementary material, figure S3). Such conversions denote Δ15 and Δ17 desaturations (table 3). Overall, these results clearly show that the *T. californicus ω*x1 holds Δ12, Δ15 and Δ17 desaturase activities. With regard to the *T. californicus ω*x2, transgenic yeast expressing its coding region were able to desaturate all C_18_ and C_20_ n–6 PUFA to n–3 PUFA products as described above for *ω*x1 but, in addition, was also able to convert 22 : 4n–6 into 22 : 5n–3 (table 3; electronic supplementary material, figure S3). These results show that the *T. californicus ω*x2 has Δ15, Δ17 and Δ19 desaturase activities.

**Table 2.** Comparison of fatty acid profiles from the transgenic yeast expressing *T. californicus ω*x desaturases with control yeast transformed with the empty pYES2 vector. The results are presented as area percentage of total fatty acids in each sample (mean ± s.e.m., *n* = 3). Asterisks (*) indicate significant differences (*p* < 0.05). n.d., not detected.

	control	*ω*x1	*ω*x2
18 : 0	8.2 ± 0.1	9.3 ± 0.1	8.7 ± 0.0
18 : 1n–9	25.2 ± 0.2	20.1 ± 0.1*	26.1 ± 0.1
18 : 2n–6	n.d.	0.8 ± 0.0*	n.d.
18 : 3n–3	n.d.	4.6 ± 0.0*	n.d.

**Table 3.** Substrate conversions of the transgenic yeast expressing the *T. californicus ω*x desaturases. The results are presented as a percentage of the fatty acid substrate converted into the corresponding desaturated product. n.d., not detected.

substrate	product	conversions (%)		activity
		*ω*x1	*ω*x2	
18 : 2n–6	18 : 3n–3	34.2	24.1	Δ15
18 : 3n–6	18 : 4n–3	43.8	25.5	Δ15
20 : 2n–6	20 : 3n–3	3.1	10.8	Δ17
20 : 3n–6	20 : 4n–3	5.0	9.7	Δ17
20 : 4n–6	20 : 5n–3	6.1	32.0	Δ17
22 : 4n–6	22 : 5n–3	n.d.	4.7	Δ19
22 : 5n–6	22 : 6n–3	n.d.	n.d.	Δ19

### 3.3. Functional characterization of the *Tigriopus californicus* front-end desaturases

**Figure 5.**
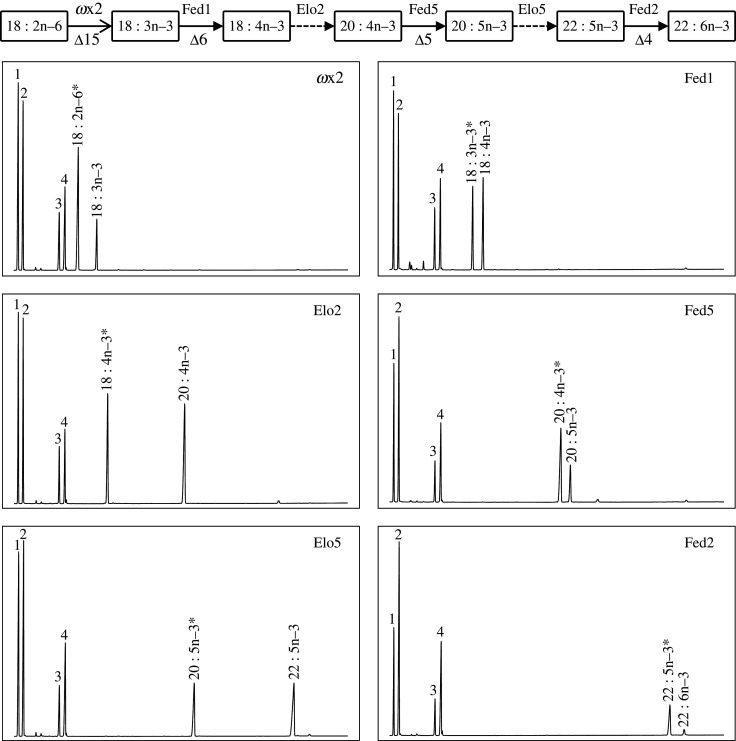
An example of the complete n–3 LC-PUFA biosynthetic pathway from 18 : 2n–6 proposed by functional analysis of the *T. californicus ω*x desaturase, front-end desaturases and fatty acyl elongases. Δy in the pathway indicates a specific carbon number from the front end of the fatty acyl chain, where the corresponding desaturase introduces a new double bond. The yeast endogenous fatty acids (16 : 0, 16 : 1 isomers, 18 : 0 and 18 : 1n–9) are indicated as 1 to 4, respectively, in all panels. Peaks corresponding to exogenously added PUFA substrates are indicated with an asterisk (*).

To characterize the functions of the *T. californicus* front-end desaturases (Fed1 to Fed5), transgenic yeast were grown in the presence of exogenously added desaturase substrates, namely n–6 (18 : 2n–6, 20 : 2n–6, 20 : 3n–6 and 22 : 4n–6) and n–3 PUFA (18 : 3n–3, 20 : 3n–3, 20 : 4n–3 and 22 : 5n–3). Our results showed that, with the exception of the Fed1 and Fed3 that share Δ6 desaturase, each front-end desaturase characterized **from* T. californicus* has a specific substrate preference. As mentioned above, Fed1 and Fed3 exhibited Δ6 desaturase activity since both enzymes were able to convert 18 : 2n–6 and 18 : 3n–3 into 18 : 3n–6 and 18 : 4n–3, respectively (figure 5 and table 4; electronic supplementary material, figure S3). Moreover, both Fed1 and Fed3 also showed Δ8 activity but, while Fed3 was able to desaturate 20 : 2n–6 and 20 : 3n–3 to 20 : 3n–6 and 20 : 4n–3, respectively, Fed1 only showed activity towards 20 : 3n–3 (table 4; electronic supplementary material, figure S3). The *T. californicus* Fed5 showed activity towards 20 : 3n–6 and 20 : 4n–3, which were converted, respectively, into the Δ5 desaturation products 20 : 4n–6 and 20 : 5n–3 (figure 5, table 4; electronic supplementary material, figure S3). The yeast expressing *Fed2* were able to convert 22 : 4n–6 and 22 : 5n–3 to 22 : 5n–6 and 22 : 6n–3, respectively, indicating that the encoded enzyme is a Δ4 desaturase (figure 5, table 4; electronic supplementary material, figure S3). No detectable activity towards the exogenously added PUFA substrates was observed for the *T. californicus* Fed 4 (table 4). None of Fed showed any detectable activity towards 24 : 5n–3 (data not shown). Furthermore, no peak corresponding to potential desaturation products of the yeast endogenous fatty acids were detected, suggesting that the *T. californicus* Fed studied herein do not possess desaturase activities towards yeast endogenous SFA and MUFA.

**Table 4.** Substrate conversions of the transgenic yeast expressing the *T. californicus* front-end desaturases (Fed1–5). The results are presented as a percentage of each fatty acid substrate converted into the corresponding desaturated product. n.d., not detected.

substrate	product	conversions (%)					activity
		Fed1	Fed2	Fed3	Fed4	Fed5	
18 : 2n–6	18 : 3n–6	30.9	n.d.	2.2	n.d.	n.d.	Δ6
18 : 3n–3	18 : 4n–3	53.7	n.d.	8.4	n.d.	n.d.	Δ6
20 : 2n–6	20 : 3n–6	n.d.	n.d.	8.8	n.d.	n.d.	Δ8
20 : 3n–3	20 : 4n–3	3.9	n.d.	13.9	n.d.	n.d.	Δ8
20 : 3n–6	20 : 4n–6	n.d.	n.d.	n.d.	n.d.	16.5	Δ5
20 : 4n–3	20 : 5n–3	n.d.	n.d.	n.d.	n.d.	28.1	Δ5
22 : 4n–6	22 : 5n–6	n.d.	10.1	n.d.	n.d.	n.d.	Δ4
22 : 5n–3	22 : 6n–3	n.d.	14.0	n.d.	n.d.	n.d.	Δ4

### 3.4. Functional characterization of the *Tigriopus californicus* fatty acyl elongases

Elongase activity was assessed by the analysis of fatty acid profiles of transgenic yeast expressing each of the *T. californicus* elongases (*Elo1* to *Elo6*) grown in the presence of exogenously added PUFA substrates, namely n–6 (18 : 2n–6, 18 : 3n–6, 20 : 4n–6 and 22 : 4n–6) and n–3 (18 : 3n–3, 18 : 4n–3, 20 : 5n–3 and 22 : 5n–3). All elongases except Elo6 showed activity towards all C_18_ and C_20_ substrates, but not towards C_22_ (figure 5, table 5; electronic supplementary material, figure S3). Particularly, low conversion efficiencies were observed for Elo1 and Elo4, with conversions often below 1.5%. Elo2 and Elo3 had relatively high conversions for all the C_18_ and C_20_ assayed (figure 5, table 5; electronic supplementary material, figure S3). Interestingly, Elo5 had relatively low elongation conversions towards C_18_ PUFA (highest 2.2% towards 18 : 3n–3) and high for C_20_ (40.6% and 56.8% towards 20 : 4n–6 and 20 : 5n–3, respectively) (figure 5, table 5; electronic supplementary material, figure S3). Elo6 was the only elongase that showed activity towards 22 : 5n–3 with low conversion efficiency (0.6%) but no activity towards 22 : 4n–6 (table 5; electronic supplementary material, figure S3). No obvious peaks corresponding to potential elongation products of the yeast endogenous FA were observed, suggesting that none of *T. californicus* Elo has elongase capacity towards yeast endogenous SFA and MUFA.

**Table 5.** Substrate conversions of the transgenic yeast expressing the *T. californicus* fatty acyl elongases (Elo1–6). The results are presented as a percentage of each fatty acid substrate converted into the corresponding elongated product. n.d., not detected.

substrate	product	conversions (%)					
		Elo1	Elo2	Elo3	Elo4	Elo5	Elo6
18 : 2n–6	20 : 2n–6	n.d.	24.7	37.3	0.7	n.d.	3.6
18 : 3n–3	20 : 3n–3	1.1	59.6	28.1	3.5	2.2	2.6
18 : 3n–6	20 : 3n–6	2.0	64.0	38.1	n.d.	n.d.	1.8
18 : 4n–3	20 : 4n–3	0.5	57.1	43.3	0.6	0.7	1.6
20 : 4n–6	22 : 4n–6	0.8	18.2	54.9	1.3	40.6	0.7
20 : 5n–3	22 : 5n–3	1.0	37.6	45.1	1.5	56.8	1.5
22 : 4n–6	24 : 4n–6	n.d.	n.d.	n.d.	n.d.	n.d.	n.d.
22 : 5n–3	24 : 5n–3	n.d.	n.d.	n.d.	n.d.	n.d.	0.6

## 4. Discussion

Copepods, one of the most abundant groups within zooplankton, contain high levels of n–3 LC-PUFA, particularly EPA and DHA [21,55], prompting interest to elucidate to which extent biosynthesis, along diet can contribute to the abundance of these essential nutrients. While several studies provided evidence suggesting that some copepod species can indeed produce LC-PUFA endogenously, the potential contribution of microbial endosymbionts for such metabolic ability could not be completely ruled out [31–33]. In the present study, we addressed this methodological drawback by performing a comprehensive retrieval for all genes encoding desaturase and elongase enzymes with potential roles in the PUFA and LC-PUFA biosynthesis in a representative species within Harpacticoida, *T. californicus*. We selected this species not only because of its importance as a dominant zooplanktonic component in the marine tidal area, but also because it has been recently shown to possess putative *ω*x desaturases, enzymes limiting the ability of animals for *de novo* biosynthesis of PUFA, as well as being major components enabling bioconversions of n–6 substrates into n–3 LC-PUFA [18]. We herein demonstrate that *T. californicus* has multiple genes encoding *ω*x desaturases, front-end desaturases and fatty acyl elongases whose functions enable this species to carry out all reactions required for *de novo* biosynthesis of PUFA and, from them, LC-PUFA up to DHA.

A comprehensive phylogenetic analysis of *ω*x desaturases performed by Kabeya *et al*. [18] depicted an overall distribution of these gene families in animals. The *T. californicus ω*x1 and *ω*x2 grouped with other copepod *ω*x desaturases to form a single clade, itself clustering along with sequences from lophotrochozoans including molluscs, annelids and rotifers [18]. In the present study, the phylogenetic analysis of front-end desaturase showed that the *T. californicus* Fed1–5 formed a well-supported branch including the other two non-Harpacticoida copepods, namely *C. rogercresseyi* (Siphonostomatoida) and *P. nana* (Cyclopoida). These results suggest that the copepod front-end desaturase genes evolved from their common ancestor. Interestingly, the front-end desaturase sequences retrieved from *T. californicus* and other copepods are phylogenetically unrelated with decapod crustacean desaturases hypothesized to be Δ6 front-end desaturases [56–60]. Unlike copepod sequences, the decapod putative front-end desaturase-like sequences do not contain the conserved Delta6-FADS-like domain (cd03506) in the NCBI-specific protein database and hence did not pass the domain-specific threshold (bit-score = 113.891) and were excluded from the ML phylogenetic inference. These findings strongly suggest that the decapod desaturases are not front-end desaturases and probably explain why no functional data supporting their annotation as ‘Δ6 desaturases’ were reported [56–60]. Indeed, the absence of front-end desaturases appears to the extent to other major groups within Malacostraca crustaceans such as isopods and amphipods, according to our search strategy that did not identify any putative front-end desaturases in these groups. It is interesting to note that the copepod front-end desaturase clade is clearly separated from the well-supported branch comprising sequences from other metazoans including vertebrates, echinoderms and lophotrochozoans (see [10,13]). This result illustrates the diversity of front-end desaturase gene families that play pivotal roles in PUFA biosynthesis in metazoans. Such diversity can be partly accounted for phenomena such as horizontal gene transfer (HGT), an evolutionary mechanism that has been proposed to explain the presence of *ω*x desaturases [18,61] and other desaturases [62] in animals. Consistently, the copepod sequences retrieved in the present study clustered closely with front-end desaturase sequences from several protists and algae including *Leishmania major* and *P. lutheri*, both characterized as Δ4 desaturases and thus playing key roles in DHA synthesis from 22 : 5n–3 [63,64]. The insufficient resolution of the tree does not allow us to conclude whether the copepod front-end desaturases were acquired via HGT. However, the ever-increasing availability of genomic sequences will probably enable us to clarify their overall evolutionary history in the near future.

The ML phylogenetic analysis of elongases denoted that four out of six genes (Elo1, Elo2, Elo3 and Elo5) isolated from *T. californicus* formed a well-supported clade with other Pancrustacea sequences. This Pancrustacea-specific clade includes the Elovl7-like elongase isolated from *S. olivacea*, which showed elongation capability on a range of PUFA [65]. The Pancrustacea elongase clade is closely related to that including the vertebrate Elovl1 and Elovl7 but, unexpectedly, not Elovl5, Elovl2 and Elovl4, major enzymes of PUFA elongation in vertebrates [13,66,67]. Moreover, the Pancrustacea elongase clade contained multiple insect sequences that, rather than roles in PUFA elongation, have been shown to participate in the biosynthesis of sex pheromones [68] and cuticular hydrocarbons [69]. Elo6 belonged to another well-supported Arthropoda clade, which includes functionally characterised Elovl4-like genes isolated from two decapod crustaceans, namely *S. olivacea* and *P. trituberculatus* [52,53] (figure 3). Unlike the other five elongase genes from *T. californicus*, one elongase gene (Elo4) clustered within the most diverged clade, which was an out-group of all other elongase sequences. Since Elo4 showed very low activity towards all PUFA tested in the present study, it is reasonable to speculate that Elo4 does not play a prominent role in PUFA elongation in *T. californicus*.

In marine ecosystems, high trophic level organisms such as fish have some capacity to biosynthesize n–3 LC-PUFA from the C_18_ n–3 PUFA ALA (18 : 3n–3), a phenomenon often referred to as ‘trophic up-grading’ [70]. Vital to this process is the provision of precursor ALA by organisms occupying lower trophic levels that serve as a food item for fish and other top predators. The functional assays of the two *T. californicus ω*x desaturases studied here showed that harpacticoid copepods can efficiently produce ALA and, hence, play such a pivotal role in marine ecosystems. Indeed, the *T. californicus ω*x desaturases contain key enzymatic activities including Δ12 (*ω*x1) and Δ15 (*ω*x1 and *ω*x2) required to convert oleic acid (18 : 1n–9) into LA (18 : 2n­–6) and ALA (18 : 3n–3), respectively (figure 1). Interestingly, the *T. californicus ω*x1, along with Δ12 and Δ15 desaturase activities, further showed Δ17 activity towards C_20_ n–6 PUFA substrates, while *ω*x2, in addition to Δ15, also had Δ17 and Δ19 activities towards C_20_ and C_22_ n–6 PUFA substrates, respectively. These results allow us to conclude that both *ω*x desaturases can be categorized as ‘*ω*3 desaturases’ enabling desaturation of multiple n–6 substrates into the corresponding n–3 metabolic products (figure 1). Similar functions were observed in one of the *ω*x desaturases functionally characterized from the Siphonostomatoida copepod *L. salmonis*, although this species possesses a further enzyme that exclusively exhibited Δ12 desaturase activity [18]. Beyond the phylogenetic diversification of desaturase gene/protein families pointed out above, these results further illustrate that diversification also expands to function since the enzymes from relatively closely related species such as *T. californicus* and *L. salmonis* have different substrate specificities.

Along with the abovementioned ability to provide C_18_ PUFA precursors for trophic upgrading to LC-PUFA by fish and other predators, our results demonstrated that harpacticoid copepods such as *T. californicus* can further produce LC-PUFA by themselves. They can do so by the combined action of the herein characterized front-end desaturases and elongases, which enable the biosynthesis of LC-PUFA through any of the routes described in animals to date (figure 1). For the *T. californicus* front-end desaturases, an apparent separation of regioselectivities was observed, with Δ6 desaturase activity being contained mostly in Fed1, Δ8 in Fed3, Δ5 in Fed5 and Δ4 in Fed2. Using the same set of enzymes in both the n–6 and n–3 routes, *T. californicus* can use two distinct pathways to biosynthesize both arachidonic acid (ARA) and EPA from the C_18_ PUFA LA and ALA, respectively (figure 1). These two pathways are the so-called ‘Δ6 pathway’, consisting of a Δ6 desaturation, followed by an elongation and a final Δ5 desaturation, and the ‘Δ8 pathway’, starting with an elongation, followed by a Δ8 desaturation and a final Δ5 desaturation (figure 1) [71,72]. In addition to front-end desaturases, we herein demonstrate that *T. californicus* has several elongases that can contribute to both pathways. More specifically, the *T. californicus* Elo2 and Elo3, with preference towards C_18_ and C_20_ PUFA, and Elo5 with preference towards C_20_ PUFA, appear to be major enzymes involved in the biosynthesis of LC-PUFA such as ARA and EPA. Although further studies will be needed to investigate physiological significance of some ‘over-lapping’ activities observed in both Fed and Elo, the apparent separation of regioselectivities/substrate specificities would imply the importance of precise modulation of LC-PUFA composition in *T. californicus*.

Copepods including harpacticoids contain lipids with particularly high levels of DHA (approximately 10 to 20%, e.g. [30,73,74]). Functional analyses of the *T. californicus* LC-PUFA biosynthesising genes demonstrate that some copepods have the ability to produce DHA from EPA. DHA biosynthesis in animals takes place via two different pathways, including the ‘Sprecher pathway’, reported in rats [75], fish [51,76,77] and the razor clam *Sinonovacula constricta* [78], and the ‘Δ4 pathway’ that is mostly present in teleost lineages [51] (figure 1). Both pathways require the first elongation from EPA (20 : 5n–3) to 22 : 5n–3 that, in the case of *T. californicus*, can be achieved by different elongases, namely Elo2, Elo3 and Elo5. However, the Sprecher pathway requires a further elongation to produce 24 : 5n–3 before this compound can be Δ6 desaturated to 24 : 6n–3 and chain-shortened (partial β-oxidation) to DHA (figure 1). None of the *T. californicus* desaturases characterized in this study appeared to be able to desaturate 24 : 5n–3 to 24 : 6n–3, and therefore it is unlikely that this copepod can produce DHA via the Sprecher pathway. Importantly, one of the *T. californicus* front-end desaturases, the herein termed ‘Fed2’ is a Δ4 desaturase, an enzyme enabling the direct conversion of 22 : 5n–3 to DHA within the Δ4 pathway. Possessing Δ4 desaturases has been previously reported in some microalgae [4] and few vertebrates mostly teleosts [22] but, to the best of our knowledge, this key enzyme has not been hitherto identified in invertebrates. While further investigations will help to clarify the occurrence of Δ4 desaturases among copepods, it is reasonable to speculate that endogenous production via the Δ4 pathway accounts for part of the DHA found in copepods' lipids [25–33].

In conclusion, we have successfully isolated and functionally characterized two *ω*x desaturases, five front-end desaturases and six elongases from *T. californicus*. The *T. californicus ω*x desaturases are *ω*3 desaturases enabling (i) the biosynthesis of LA and ALA that are precursors of LC-PUFA and (ii) the conversion of multiple n–6 PUFA into the corresponding n–3 metabolic products including LC-PUFA. Upgrading from the C_18_ PUFA LA and ALA to LC-PUFA is also possible in *T. californicus*, since the complementary action of its front-end desaturases and elongases enable multiple routes for the biosynthesis of the physiologically active compounds ARA, EPA and DHA. Collectively, the results obtained in this study demonstrate that *T. californicus* has a complete enzymatic complement enabling this species to produce n–3 LC-PUFA up to DHA endogenously. Therefore, harpacticoid copepods arise as primary producers of n–3 LC-PUFA in marine ecosystems and, given their widespread distribution and abundance at a global scale, it is likely that such contribution is not negligible.

## Data accessibility

All isolated sequences from *T. californicus* in the present study were deposited into the NCBI GenBank with the accession numbers MT757162 to MT757173 and MW246081.
